# High Expression of Cry1Ac Protein in Cotton (*Gossypium hirsutum*) by Combining Independent Transgenic Events that Target the Protein to Cytoplasm and Plastids

**DOI:** 10.1371/journal.pone.0158603

**Published:** 2016-07-08

**Authors:** Amarjeet Kumar Singh, Kumar Paritosh, Uma Kant, Pradeep Kumar Burma, Deepak Pental

**Affiliations:** 1 Centre for Genetic Manipulation of Crop Plants, University of Delhi South Campus, Benito Juarez Road, New Delhi-110021, India; 2 Department of Genetics, University of Delhi South Campus, Benito Juarez Road, New Delhi-110021, India; National Key Laboratory of Crop Genetic Improvement, CHINA

## Abstract

Transgenic cotton was developed using two constructs containing a truncated and codon-modified *cry1Ac* gene (1,848 bp), which was originally characterized from *Bacillus thuringiensis* subspecies *kurstaki* strain HD73 that encodes a toxin highly effective against many lepidopteran pests. In Construct I, the *cry1Ac* gene was cloned under FMVde, a strong constitutively expressing promoter, to express the encoded protein in the cytoplasm. In Construct II, the encoded protein was directed to the plastids using a transit peptide taken from the cotton *rbcSIb* gene. Genetic transformation experiments with Construct I resulted in a single copy insertion event in which the Cry1Ac protein expression level was 2–2.5 times greater than in the *Bacillus thuringiensis* cotton event Mon 531, which is currently used in varieties and hybrids grown extensively in India and elsewhere. Another high expression event was selected from transgenics developed with Construct II. The Cry protein expression resulting from this event was observed only in the green plant parts. No transgenic protein expression was observed in the non-green parts, including roots, seeds and non-green floral tissues. Thus, leucoplasts may lack the mechanism to allow entry of a protein tagged with the transit peptide from a protein that is only synthesized in tissues containing mature plastids. Combining the two events through sexual crossing led to near additive levels of the toxin at 4–5 times the level currently used in the field. The two high expression events and their combination will allow for effective resistance management against lepidopteran insect pests, particularly *Helicoverpa armigera*, using a high dosage strategy.

## Introduction

Cotton (*Gossypium hirsutum*) is an important fiber crop that is grown extensively in many parts of the world. Apart from providing very valuable fiber, cotton seed is used as a source of edible oil and seed cake, which is used as animal feed. In 2014, ~37 million hectares of land was sown with cotton worldwide [1], mostly under rain fed and dryland conditions. In India alone, the crop was grown in ~12.25 million hectares of land [1].

Cotton is highly susceptible to a large number of lepidopteran pests, and a major one on the Indian subcontinent is *Helicoverpa armigera*. A milestone in cotton breeding was the development of transgenic pure-line varieties and hybrids containing the *cry* genes of *Bacillus thuringiensis* (*Bt*) encoding insecticidal proteins that provide protection from lepidopteran pests [2]. Globally, ~68% percent of the cotton crop is transgenic, containing *Bt* genes and/or resistance to herbicide glyphosate. In 2014–15, ~95% (11.6 million hectares) of the area producing cotton in India was sown with transgenic hybrids containing the *cry1Ac* gene (Bollgard I) or two *cry* genes, *cry1Ac* and *cry2Ab*2 (Bollgard II). Both Bollgard I and Bollgard II utilize a *cry1Ac-like* gene construct that was originally present in the event Mon 531 [2]. Since the introduction of *Bt* cotton in India, the cotton production area has increased from 7.7 million hectares to 12.25 million hectares, fiber production has increased from 13.6 million bales to 39.1 million bales, and pesticides usage to control lepidopteran pests has decreased from 5,748 metric tons to 222 metric tons [3]. A number of studies have shown that *Bt* cotton has increased farmer’s incomes, including those of smallholder farmers, reduced pesticide usage and even improved natural biocontrol [4–9].

A major challenge for transgenic cotton is the management of resistance development in insect pests feeding on the crop, also called insect resistance management, which has garnered considerable attention [2, 10–14]. The first release of transgenic cotton stipulated the use of a refuge of non-transgenic cotton plants and also of insecticidal prophylactic sprays to manage the development of resistant variants of the target pests [2]. Out of a number of strategies for insect resistance management, three are key–(i) achieving high toxin dosage either by the use of strong promoters [2, 15], or by targeting the protein to organelles [16, 17] or by tissue specific expression of the protein [18]; (ii) use of multiple genes [19, 20, 21] preferably, those that work through different mechanisms [22, 23] and (iii) use of a refuge along with (i) and (ii) [10, 11, 24]

A major weakness of the products (Bollgard I and II) currently used in the field is a drop in the Cry1Ac protein’s expression level as the plant matures and sets bolls [25, 26, 27]. Further, there is a high expression level in the roots that provides no resistance against *H*. *armigera* and other lepidopteran pests, as they do not feed on roots. Another weakness is that a secondary lepidopteran pest on cotton, *Pectinophora gossypiella*, can survive the low Cry1Ac protein dose present in the developing bolls of event Mon 531 [28, 29] and leave progeny.

We report our results on developing transgenic events in cotton that express the Cry1Ac protein at levels higher than the products presently used in the field. We studied a transgenic event that resulted in the Cry1Ac protein being accumulated in the cytoplasm, and an event in which the Cry1Ac protein is targeted to the plastids. Additionally, we show that when these two events are combined sexually, the progeny show near additive levels of the Cry1Ac protein. This strategy could be of use in developing high-toxin dosage plants to manage resistance development more effectively. Our results also conclusively show that a transgenic protein having the transit peptide of a protein that accumulates in green plastids does not get targeted to the leucoplasts. Using such a transit peptide could be an effective method for expressing a transgene-encoded protein only in green aerial tissues.

## Materials and Methods

### Gene constructs for genetic transformation

**Fig 1:**
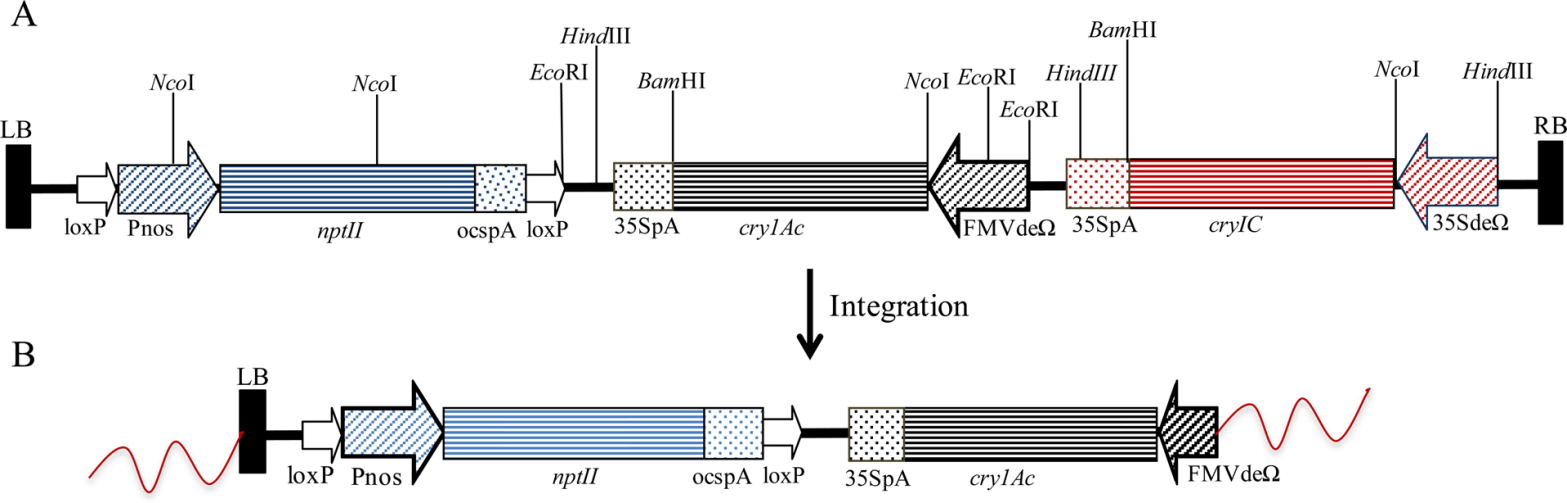
Construct I and its state of integration in the genome of event Tg2E-13. (A) Schematic representation of the T-DNA region of Construct I; (B) Integrated pattern of T-DNA cassettes in the genome in the transgenic event Tg2E-13 as discerned by genome walking experiments. Only two of the transgene cassettes contained in the construct–namely *nptII* gene and *cry1Ac* gene have integrated in the plant genome.

**Fig 2:**
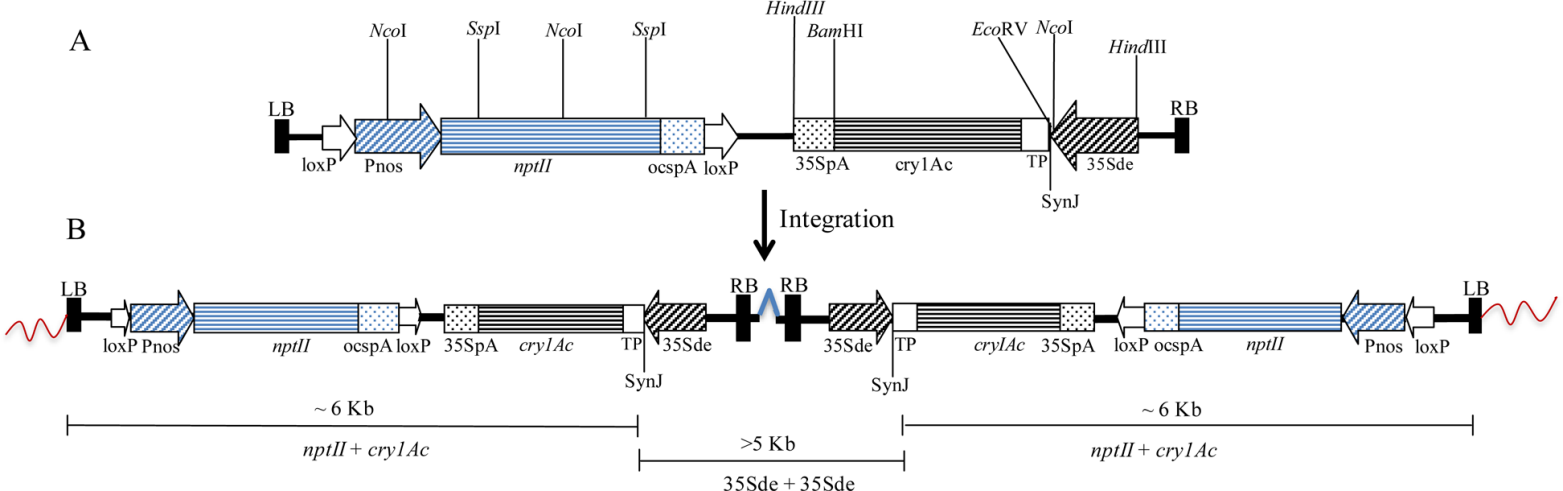
Construct II and its state of integration in the genome of event TM-2. (A) Schematic representation of the T-DNA region of Construct II; (B) Pattern of integration of the T-DNA region in the transgenic event TM-2 as discerned by genome walking experiments. Two copies of the transgene cassettes (T-DNA region) are present in an inverted manner.

Two different constructs were used for the genetic transformation of cotton. Construct I contained three gene expression cassettes–Pnos-*nptII*-ocspA::FMVde-Ωleader-*cry1Ac*-35SpA::35Sde-Ωleader-*cry1C*-35SpA. Construct II contained two gene cassettes–Pnos-*nptII*-ocspA::35Sde-synJUTR-TP-*cry1Ac*-35SpA. In both the constructs, the nos-*nptII* gene cassette, used for *in vitro* selection, was cloned within LoxP sites for the eventual removal of the marker gene by crossing the *cry* gene-containing transgenic lines with cotton lines containing the *cre* gene [30]. The *cry1Ac* gene used for developing transgenics was synthesized by overlapping oligos and recursive PCR in the lab [31], and its nucleotide sequence is different from the *cry1Ab-cry1Ac* fusion gene [32] present in the event Mon 531, which has been deployed worldwide. The sequence used here encodes a protein with an amino acid sequence that is identical to that described by Adang et al. [33]. A comparison of the nucleotide and the encoded protein sequences of the *cry1Ac* gene used in this study, as compared with cry1Ac variant currently in use in India and elsewhere, is provided in the S1 Appendix (nucleotide sequences) and S2 Appendix (protein sequences). Constructs used for genetic transformation have been shown in Figs 1A and 2A. In Construct I, the codon modified *cry1Ac* gene was cloned under a FMV double enhancer promoter [34] with a 5' Ω leader (UTR) sequence [35] cloned between the promoter and the translation start site. A third gene cassette included a *cry1C* gene of ~2 kb [36] under the control of the CaMV35Sde promoter [37] and a CaMVpA site at the 3' end of the coding region (Fig 1A). In Construct II, the codon-modified *cry1Ac* gene sequence (~1.8 kb) was cloned under a CaMV35S double enhancer promoter. A transit peptide sequence from the cotton *rbcS1b* gene [38] was cloned between synJ UTR [39] and the *cry1Ac* gene sequence to create a reading frame between the transit peptide and Cry1Ac protein-encoding sequence (Fig 2A). Gene cassettes were cloned within the LB and RB of the *Agrobacterium* binary vector pPZP200 [40].

### Plant materials and genetic transformation

The *G*. *hirsutum* Coker 310FR line developed earlier [41] was used for genetic transformations in all of the experiments. Sterilized seeds were placed on ½ MS medium at 28°C ± 2°C, with 1,000 lux light intensity and a 16-h light/8-h dark photoperiod for seven days. Cotyledonary tissues of seven-day-old seedlings were used for genetic transformation. Genetic transformation and *in vitro* culturing were carried out as described earlier [42]. *In vitro* regenerated plants were grown in a growth chamber at 30/26°C ± 2 day/night temperature, with 2,000 lux light intensity and a 16-h light/8-h dark cycle at a relative humidity of ~80%.

### Genomic DNA isolation and Southern blot analysis

Genomic DNA was isolated from cotton leaves using a modified version of the CTAB method [43]. DNA was purified by ‘DNeasy Plant Maxi Kit’ (Qiagen) for PCR and other molecular analyses.

For the Southern blot analysis, ~10 μg of DNA isolated from each line was digested with appropriate restriction enzymes. λ DNA digested with *Hind*III was used as a size marker. Digested DNA samples were electrophoresed on a 0.8% agarose gel and blotted on a nylon membrane (Hybond N+; Amersham Pharmacia Biotech). The presence of the different gene cassettes and their integration patterns in the transgenics were studied using appropriate probes. Probes were labeled with α-[^32^P]–dCTP using a ‘Megaprime DNA Labeling System’ (Amersham Pharmacia Biotech). Standard procedures were followed for hybridization and washing. Membranes were subjected to autoradiography for 36–48 h at -80°C.

PCR amplification reactions to detect the presence of gene sequences were performed with gene-specific primers (S3 Appendix) using 50 ng of DNA in a reaction volume of 25 μl. The reaction mix comprised 200 μM dNTPs, 12.5 pmol of specific primers, 2.5 U of Taq polymerase, and 1× Taq buffer. The PCR conditions used were as follows: initiation with a 5 min denaturation at 95°C, followed by 30 amplification cycles of 30s at 95°C, 30s at 65°C and 1 min at 72°C. A final extension was performed at 72°C for 5 min.

### Cloning of plant genome fragments flanking the T-DNA inserts by genome walking

To develop libraries for genome walking, DNA isolated from two of the events was purified by CsCl density gradient centrifugation. Genomic libraries were constructed using a ‘Genome Walker Kit’ (Clontech) following a previously described protocol [38]. Six different libraries were developed using six different restriction enzymes, *EcoR*V, *Hinc*II, *Ssp*I, *Sca*I, *Msc*I and *Xmn*I. Restriction enzyme fragments were ligated to a universal adaptor. The right border upstream region of the gene cassette was amplified using *cry1Ac* gene-specific nested reverse primers and adapter-specific forward primers, while for the left border flanking sequences, nested primers from the *nptII* gene were used in combination with adapter-specific nested primers (S3 Appendix). Amplification reactions were carried out using ‘Takara HS Polymerase’ (Takara). Amplified fragments were resolved on agarose gels, eluted and cloned in ‘pGEM-T Easy Vector’ (Promega), and sequenced from both ends. Final consensus sequences were derived from three independent PCR reactions. Consensus sequences were aligned with the gene cassette and cotton gene sequences [44] using DNASTAR software.

### Detection of the Cry1Ac protein in transgenic plants and statistical analysis

The T_0_ generation of transgenic events was grown in a growth chamber. Subsequent generations were grown in soil in a containment net house. Five independent plants of each line were used for analysis. Expression of the Cry1Ac protein was studied in the leaf tissues from 20-, 30-, 60-, 90-, 100- and 120-day-old plants, and cotyledons and root tissues from 15-day-old plants. On each sampling date, a fully expanded leaf from the apex was used for the analysis. However, the Cry1Ac expression analysis in 20-day-old plants was carried out using a whole young leaf. Samples were collected from the field and transported to the laboratory on ice.

Two different ‘ELISA Kits’ (Envirologix), qualitative and quantitative, were used. The ‘Qualitative ELISA Kit’ was used to screen Cry1Ac positive plants, whereas the ‘Quantitative ELISA Kit’ was used to measure the amount of Cry1Ac protein in different organs and tissues. The quantitative ELISA test is based on a double-antibody sandwich enzyme-linked immunosorbent assay. For the extraction of total protein, whole young leaves of 20-day-old plants, flower buds, bract and root tissues, after being weighed, were ground in a mortar with 1 ml of the extraction buffer provided in the kit. In case of 30-, 60-, 90-, 100- and 120-day-old plants and cotyledons of 15-day-old seedlings, ~40 to 45 mg of leaf and cotyledon tissues were ground in 1 ml of the extraction buffer. The resulting extracts were centrifuged and the total soluble protein content present in the cell-free extracts was determined by a dye-binding procedure using bovine serum albumin as a standard protein. The ELISA was performed according to the manufacturer’s instructions (Envirologix). The quantification of the Cry1Ac protein was carried out by plotting absorbance values of Cry1Ac test samples onto the standard curve generated by using the purified Cry1Ac protein. The quantity of the Cry1Ac protein was measured in μg/g fresh weight of test samples. Each sample was analyzed in duplicate. Expression levels of the Cry1Ac protein present in the transgenic events were compared with the expression levels of the Cry1Ac protein in the two commercialized lines BioCot-1 (hemizygous for the event Mon 531) and BioCot-2 (homozygous for the event Mon 531). BioCot-1 and BioCot-2 seeds were provided by Shriram Bioseed Genetics, India.

The normality of the expression data’s distribution was determined using the Kolmogorov-Smirnov test. Differences at the *P* < 0.05 level were considered statistically significant. Observed expression values were compared separately with the normally distributed dataset having the same mean and standard deviations. A *P* > 0.05 was recorded for each of the checked datasets, which indicated that the observed distribution was not significantly different from the normally distributed dataset. All of the analyzed samples were found to be normally distributed. The expression values for the Cry1Ac protein levels at different growth time points were log2 transformed and subjected to a one-way ANOVA test. The means obtained for different events were compared using Tukey’s HSD method. All of the statistical analyses were conducted using the R software package.

### RNA isolation and RT-PCR analysis

For the RT-PCR analysis, total RNA was isolated from the root and cotyledons of plants using the ‘Spectrum Plant RNA Isolation Kit’ (Sigma) following the manufacturer’s instructions. Isolated RNA was treated with DNaseA (DNA-*free* Kit, ABI). cDNA synthesis was carried out from 1 μg of total RNA using the ‘High Capacity cDNA Reverse Transcriptase Kit’ (ABI) with random hexamers in a 30 μl reaction volume. Then, 2 μl of the cDNA pool was used for real time quantification of *cry1Ac*, as well as the 18S rRNA as a control gene, using TaqMan probes and primers [45] (S3 Appendix). PCR reactions were set with 1× ‘TaqMan Master Mix’ (ABI), 9 pmol of each primer and 2.5 pmol of probes in a 10 μl reaction. qPCR was performed on ‘QuantStudio 6 Flex Real-Time PCR System’ (Thermo Fisher).

### Insect Bioassays

*H*. *armigera* larvae were reared on a chickpea-based artificial diet [46] in a room set at 27 ± 1°C, with a relative humidity of 65 ± 5% and a 16-h light/8-h dark cycle. Four-day-old larvae were used for assessing feeding behavior on leaf tissues of the selected transgenic lines. Leaf pieces taken from different transgenic lines were kept inside petri dishes on a moistened filter paper. For insect feeding bioassays, a single four-day-old larva was released in each petri dish to assess the sensitivity of *H*. *armigera* to the protein encoded by the transgene in the leaf tissues. A total of 30 larvae were used for each experiment.

## Results

### Genetic transformation with Construct I and selection of event Tg2E-13

Cotyledonary explants of the Coker 310FR line were used for *Agrobacterium*-mediated genetic transformation with Construct I (Fig 1A). A total of 1,586 explants were cultured *in vitro*, of which only 18% produced embryogenic calli on the selection medium, and embryos were recovered from embryogenic calli at a frequency of 6.2%. A total of 36 putative transgenic lines were grown in soil. Out of the putative lines, 22 were observed to be phenotypically abnormal (Table 1). All of the lines were subjected to a qualitative ELISA to identify plants positive for the Cry1Ac protein. In total, 25 lines showing Cry1Ac expression were analyzed to determine the insert copy number by Southern blot hybridization using *cry1Ac* and *nptII* gene fragments as probes (S4 Appendix). Two events, Tg2E-13 and Tg2H-8, were selected for further analysis as these were single copy events that exhibited normal growth. Transgenic lines were selfed to generate T1 generation seeds. The selfed seeds (T1) of the two events were grown in ½ MS medium containing 100 mg/l kanamycin. Kan^r^ progeny of the two events were grown in a growth chamber. In Cry1Ac expression studies, Mech 12 and Bollgard II, which both contain the *cry1Ac* gene of the event Mon 531, were used as positive controls and Coker 310FR was used as a negative control. Because the Tg2H-8 line had a very low Cry1Ac protein expression level in the T1 generation (results not shown), this line was not analyzed further. Only progeny of the Tg2E-13 event showed high Cry1Ac protein expression levels. Based on these analyses, event Tg2E-13 was selected for further assessment in the advanced generations.

**Table 1:** Frequency of embryogenic callus formation, somatic embryos and recovery of putative transgenics following transformation of Coker 310FR with Construct I and Construct II.

Constructs	No of Explants	Embrogenic calli as % of explants	Embryos as % of explants	Plantlets as % of explants	Qualitative ELISA		Phenotype	
					Cry1Ac positive	Cry1Ac negative	Normal	Abnormal
Pnos-*nptII*-ocspA::FMVde-Ωleader-*cry1Ac*-35SpA::35Sde-Ωleader-*cry1C*-35SpA.	1586	18	6.2	2.3	25	11	14	22
Pnos-*nptII*-ocspA::35S-synJUTR-TP-*cry1Ac*-35SpA	2714	26.5	7.7	2.6	27	43	61	9

### Characterization of event Tg2E-13

Event Tg2E-13 was backcrossed and selfed for four generations, and the expression of the Cry1Ac protein was monitored in each generation. Event Tg2E-13 was a high Cry1Ac expression event that was free from any abnormalities. A line homozygous for this event was developed using a progeny analysis of the T2 generation plants. Assessment of transgene stability and Cry1Ac protein expression patterns was carried out under confined conditions in a growth chamber or under field conditions in a containment net house. Southern blot analysis was repeated using a number of probes. Event Tg2E-13 contained a single copy of the *cry1Ac* gene and also contained the marker gene cassette, but it did not contain any sequence of the *cry1C* gene. Southern analysis of the Tg2E-13 event is fully described in S4 Appendix.

Genome walking was performed in Tg2E-13 from *nptII* and *cry1Ac* gene sequences. A perfect match was found between the *cry1Ac* gene cassette and the amplified walk DNA fragments. Only 167 bp of the FMV promoter region out of the 564-bp cloned sequence was present upstream of the *cry1Ac* gene and, thereafter, the *G*. *hirsutum* genomic flanking sequence was present (Fig 1B). Sequencing data clearly showed that the region on the right border of the expression cassette, encompassing part of the FMV promoter and the *cry1C* gene cassette had been deleted. The BLASTN algorithm was used to search for available cotton genome sequences [47, 48] and showed similarities to the sequences from chromosome D9 of the *G*. *hirsutum* genome. With the walk from the *nptII* gene sequences, a perfect match was found until the left border sequence, except a deletion of ~80 bases in the upstream region of the nos promoter. The left border flank also showed similarity to the sequences on chromosome D9 of the cotton genome. Genome walking results confirmed the Southern hybridization results on the presence of a truncated FMV promoter and the absence of *cry1C* cassette from the integrated DNA.

### Genetic transformation with Construct II and selection of event TM-2

Construct II (Fig 2A) was used to transform 2,714 cotyledonary explants. Of these, 26.5% produced embryogenic calli, and embryos were recovered from embryogenic calli at a frequency of ~7.7%. A total of 70 putative transgenic lines were generated. Of these, 61 lines were phenotypically normal (Table 1). Qualitative ELISA was carried out for screening Cry1Ac positive plants. A total of 27 lines showed the expression of the Cry1Ac protein. Positive transgenic events were selfed to generate T1 seeds. A total of 22 plants were selected for Southern hybridization analysis (S5 Appendix), and expression levels of the Cry1Ac protein were compared with expression levels observed in the commercial lines containing the *cry1Ac* gene. Three events, RA-2, RD-8 and TM-2, were selected for expression analysis of the Cry1Ac protein based on the copy number of the integrated T-DNA and a normal phenotype. Selfed seeds (T1) of the three events were grown in ½ MS medium containing 100 mg/l kanamycin. Kan^r^ progeny of these events were grown in a growth chamber. For expression studies, BioCot-1 and BioCot-2 were used as positive controls, while Coker 310FR was used as a negative control. Expression studies were performed on the leaf tissues at different growth time points. Progeny of event TM-2 showed higher expression Cry1Ac protein levels compared with the progeny of the RA-2 and RD-8 events at different stages of growth and development (data not shown). Based on the preliminary results, event TM-2 was selected for a more detailed analysis.

### Characterization of event TM-2

Event TM-2 was analyzed over three generations for its phenotypic stability and Cry1Ac protein expression levels. The latter was checked both from plants grown in a growth chamber and under open conditions in a containment net house. A Southern blot analysis showed that this event contained two expression cassettes at a single locus in an inverted fashion (Fig 2B; S5 Appendix). An analysis of the flanking sequence by genome walking confirmed this observation.

Genome walking experiments on event TM-2-containing transgenic plants showed two different types of sequences from the left border genome walk, indicating that two copies of the *nptII* cassette were present in the genome. These two different sequences were obtained after performing walk reactions from the *cry1Ac* gene, which showed a complete sequence until the right border and after that flanking genome sequences were obtained. The BLASTN algorithm was used to search with available cotton genome sequence [47, 48]. The flanking regions at both borders showed sequence similarities to chromosome A11 of cotton. The analysis indicates that two copies of the gene cassette were integrated onto chromosome A11 of the cotton genome in an inverted fashion (Fig 2B).

### Expression analysis of Cry1Ac protein in transgenic events Tg2E-13 and TM-2

**Fig 3:**
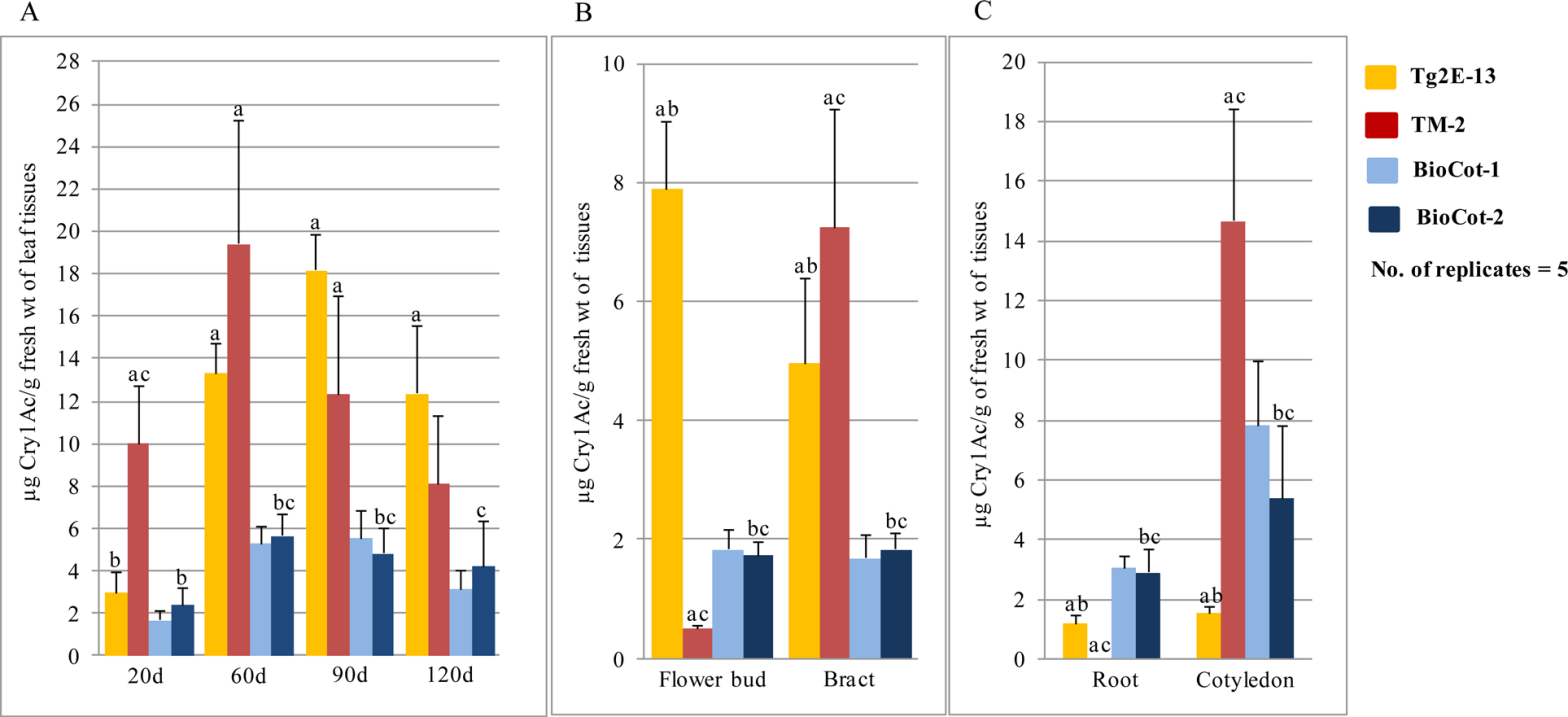
Levels of the Cry1Ac protein in μg/g fresh weight (Mean±SD) in different tissues of the two transgenic events of cotton developed in this study, Tg2E-13 and TM-2, and their comparison with two commercial lines—BioCot-1 containing *cry1Ac* gene of the event Mon 531 in a hemizygous condition and BioCot-2 containing the *cry* gene in a homozygous condition; data is from the growing season of 2014. (A) Cry1Ac expression in the leaf tissues of 20, 60, 90 and 120 day old plants; (B) Cry1Ac expression in the flower bud and bract tissues of 70 day old plants; (C) Cry1Ac expression in the root and cotyledonary tissues of seedlings. Significantly different expression values (*P* >0.05) are denoted with letters above each of the bar. Letter “a” “b” and “c” denote significantly different values as compared to the values in BioCot-2, TM2 and Tg2E-13 lines, respectively.

Although a quantitative ELISA was performed on the selected events in every generation, a more involved comparative analysis of Cry1Ac protein expression was carried out in 2014 and 2015 during the normal cotton growing season on plants grown in the field in a containment net house. Homozygous seeds of event Tg2E-13 and hemizygous seeds of event TM-2 were used to quantify the Cry1Ac protein present in the leaf tissues of 20-, 30-, 60-, 90-, 100- and 120-day-old plants in 2014. The toxin levels declined with the age of the plant, and the maximum expression levels of events Tg2E-13 and TM-2 occurred in 90- and 60-day-old plants, respectively. In comparison, not much difference was observed in the Cry1Ac protein expression levels of the two BioCot lines in 60- and 90-day-old plants. However, most importantly, the mean Cry1Ac protein expression levels in leaf tissues taken at different growth time points were 2- to 3-fold and 3- to 4-fold higher in events Tg2E-13 and TM-2, respectively, at every stage than the expression levels observed in the two BioCot lines containing the event Mon 531 (Fig 3A). Further, a one-way ANOVA analysis with Tukey’s HSD test at *P* < 0.05 showed that the mean expression levels of the Cry1A protein in the events Tg2E-13 and TM-2 plants were significantly different than those of BioCot lines in the leaf tissues at different time points.

To evaluate the spatial expression of the Cry1Ac protein, flower buds and bracts were subjected to ELISA-based quantitative assays in Tg2E-13 and TM-2 events and in controls. For event Tg2E-13, the Cry1Ac protein expression level was variable in different plant parts. The maximum occurred in the flower bud tissues, followed by in the bract tissues (Fig 3B). A one-way ANOVA followed by Tukey’s HSD test showed that the amount of Cry1Ac protein was significantly higher in the flower buds of event Tg2E-13 than in the BioCot lines, while the amount of Cry1Ac protein in event TM-2 was significantly lower than in the BioCot lines (*P* < 0.05). However, both the Tg2E-13 and TM-2 events showed significantly higher Cry1Ac protein levels when compared with the protein levels in the bract tissues of the BioCot lines (*P* < 0.05). The Cry1Ac protein expression levels in the flower buds were ~4-fold higher than those of the BioCot-1 and BioCot-2 lines. In bract tissues, the expression levels in Tg2E-13 and TM-2 were ~3-fold higher than in BioCot-1 and BioCot-2. For event TM-2, the maximum expression level was observed in the bract tissue, followed by in the flower bud tissues (Fig 3B). The expression level of the Cry1Ac protein in the flower buds of event TM-2 was around 4-fold less than in the BioCot-1 and BioCot-2 lines. However, the bract tissue had a 4-fold higher expression level when compared with expression levels in similar tissues of the BioCot-1 and BioCot-2 lines.

Interestingly, in event Tg2E-13, the Cry1Ac protein’s expression level was observed to be ~3-fold less in the root tissues and ~4-fold less in the cotyledonary tissues when compared with the BioCot lines. In event TM-2, the Cry1Ac protein’s expression level was ~2-fold higher in the cotyledonary tissues when compared with the BioCot lines. However, no expression of the protein was recorded in the root tissues in event TM-2-containing plants (Fig 3C). All the recorded differences were highly significant as revealed by a one-way ANOVA followed by Tukey’s HSD analysis, except that no Cry1Ac protein was detected in the root tissues of event TM-2 lines (Fig 3A, 3B and 3C; S7 Appendix).

### Crosses of lines containing the events Tg2E-13 and TM-2 resulted in near additive Cry1Ac protein expression in the progeny

**Fig 4:**
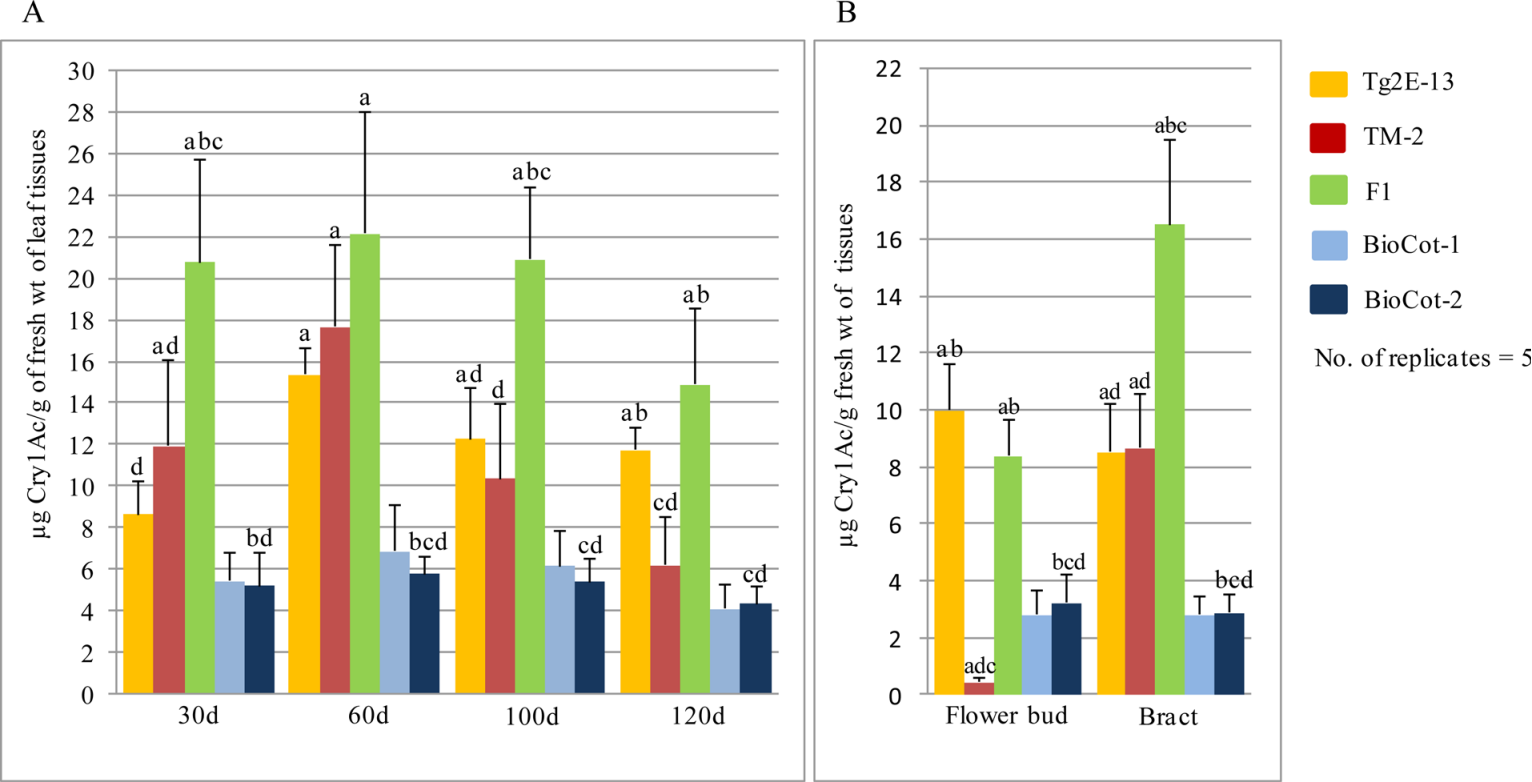
A comparative study of expression levels of the Cry1Ac protein in μg/g fresh weight (Mean±SD) in the leaf and other tissues of transgenic events Tg2E-13, TM-2 and their F1 (Tg2E-13 x TM-2); results are from the growing season of 2015. (A) Cry1Ac expression in leaf tissues of 30, 60, 100 and 120 day old plants; (B) Cry1Ac expression in the flower bud and bract tissues of 70 day old plants. Significantly different expression values (*P*>0.05) are denoted with letters above each of the bar. Letter “a” “b”, “c” and “d” denote significantly different values as compared to the values of BioCot, TM2, Tg2E-13 and F1 plants, respectively.

A line homozygous for event Tg2E-13 was crossed with a line hemizygous for event TM-2 to obtain F1 seeds. The progeny plants were checked for the presence of the two expression cassettes using PCR-based amplification reactions that could discriminate between the *cry1Ac* cassettes in Constructs I and II (S6 Appendix). Progeny plants containing both the gene cassettes were found to have normal phenotypes. Cry1Ac protein expression studies in plants containing the events Tg2E-13, TM-2 and their F1 progeny were performed in a containment net house during the 2015 growing season. The expression level of the Cry1Ac protein was quantified in leaf tissues, flower buds and bracts at different time points. Very high Cry1Ac protein expression levels were observed in leaves, bracts and flower buds (Fig 4A and 4B). The mean expression levels in the F1 plants were found to be significantly higher than in TM2, Tg2E-13 and the BioCot lines at most of the analyzed time points according to a one-way ANOVA and subsequent Tukey’s HSD test at *P* < 0.05 (Fig 4A and 4B; S7 Appendix). The expression levels in the progeny were found to be additive in comparison with expression levels recorded in the events Tg2E-13 and TM-2 at the corresponding stages, even though the two Cry1Ac-containing cassettes were present in a hemizygous condition in the F1 plants. The Cry1Ac protein’s expression level did not decline even in the leaves of 120-day-old plants to the extent that occurred in Tg2E-13 and TM-2 plants individually. The overall expression level of the Cry1Ac protein in the F1 (Tg2E-13 × TM-2) were ~4- to 5-fold higher than in the commercial *Bt*-cotton varieties tested in the study.

### Analysis of the Cry1Ac-encoded mRNA in the transgenic events Tg2E-13 and TM-2

**Fig 5:**
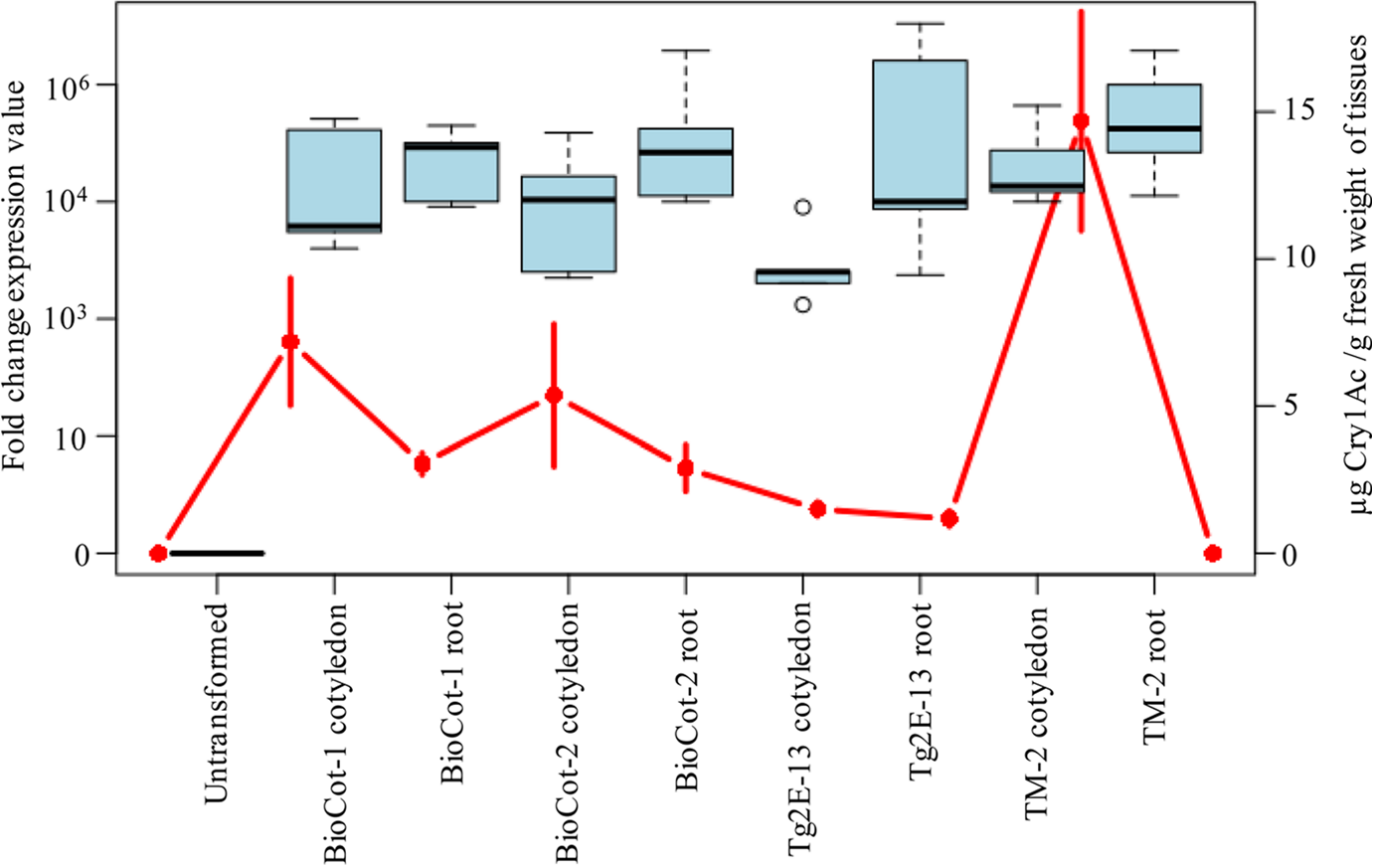
Transcript levels of the *cry1Ac* gene and the encoded protein in the events Tg2E-13 and TM-2. Comparative mRNA levels of *cry1Ac* gene in the cotyledon and root tissues of seedlings of the two transgenic events Tg2E-13, TM-2 and commercialized lines BioCot-1 and BioCot-2. Five biological replicates were used for each of the sample tissue. Similar age tissues from the same plants were used for quantification of the mRNA and the protein encoded by the *cry1Ac* gene. The fold change in transcript levels are represented in box and whisker plot while the protein levels (Mean±SD) are represented as red dots. As expected, no mRNA expression was recorded in the control tissues. Expression of the *cry1Ac* encoded mRNA was high in the root tissues of event TM-2 but the amount of Cry1Ac protein was zero like in the control plants.

The CaMV 35S promoter drives high expression of genes cloned downstream to it in the root tissues [49, 50]. However, we observed no expression of the Cry1Ac protein in the root tissues of event TM-2. To analyze whether this lack of expression was due to an absence of mRNA transcripts or the degradation of the transgenic protein, we performed a real-time transcript analysis of the *cry1Ac* gene in the events TM-2 and Tg2E-13. Transcripts were quantified in the cotyledons and root tissues of at least five plants harboring one of the two events. The level of the *cry1Ac* transcript in cotyledons and root tissues was high in both events, while, as expected, no transcripts specific to the *cry1Ac* gene were recorded in the untransformed control plants (Fig 5). *cry1Ac* transcript levels more or less corresponded to the protein levels in the cotyledonary and root tissues. For the event TM-2, abundant *cry1Ac* transcripts were found in the root tissues and the cotyledons, but the level was higher in the roots than in the cotyledons (Fig 5). However, as observed in the 2014 growing season, no protein encoded by the *cry1Ac* gene was found in the non-green root tissues of event TM-2-containing plants. In contrast, high levels of the Cry1Ac protein were present in the green cells that contained mature plastids (Fig 5).

### Toxicity assessment of selected transgenic events on *H*. *armigera*

**Fig 6:**
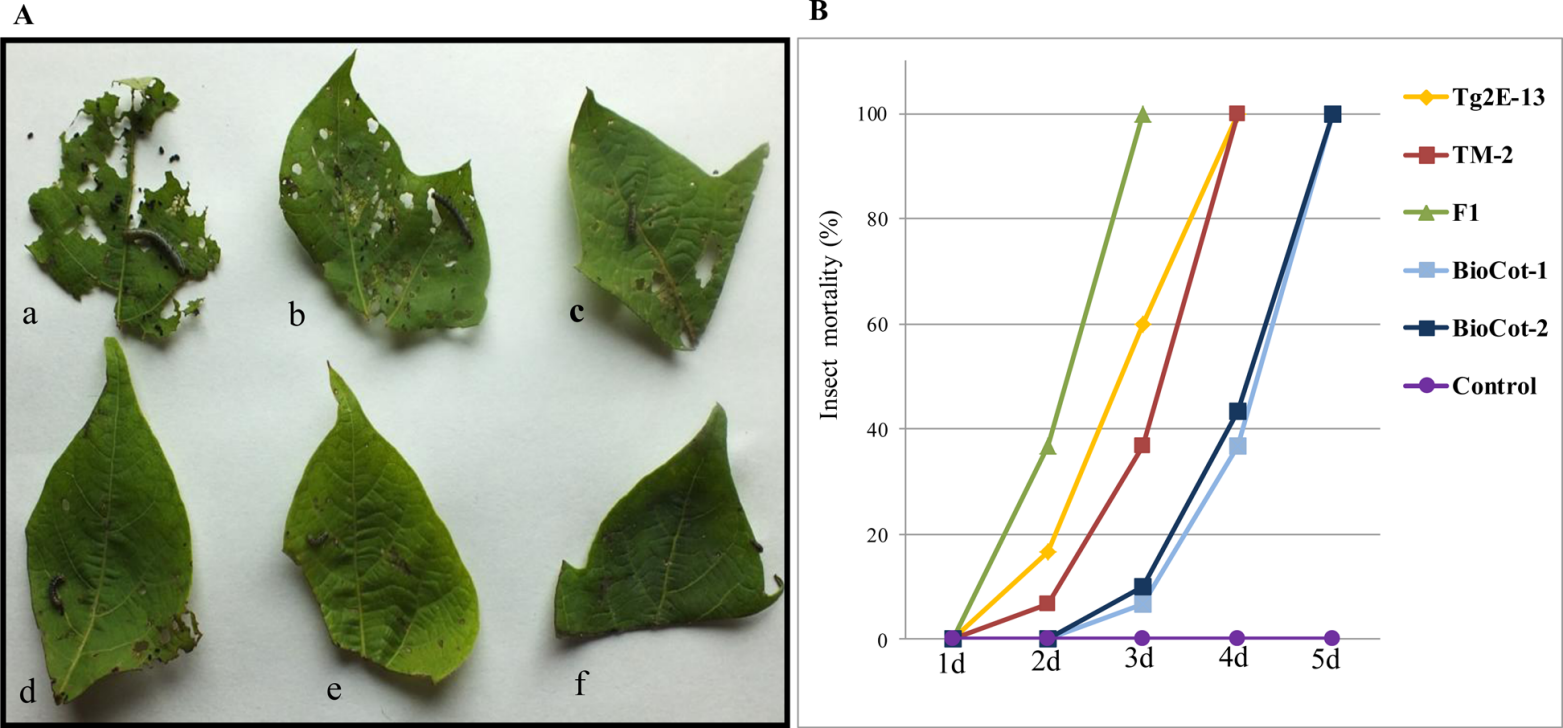
Insect bioassays using leaf tissues of l20 day old control and transgenic lines of cotton. Four day old insect larvae were released on the leaf tissues and pictures were taken after 3 days of feeding. (A) Consumed surface area in (a) the control plant (b) BioCot-1 (event Mon 531 in a hemizygous condition) (c) BioCot-2 (event Mon 531 in a homozygous condition) (d) eventTM-2 (e) eventTg2E-13 and (f) F1 (TM-2 x Tg2E-13); (B). Percent mortality of larvae fed on leaf tissues of different transgenic lines.

Insect toxicity assays were carried out on *H*. *armigera* using leaf materials from transgenic plants containing the events Tg2E-13, TM-2, their F1(Tg2E-13 × TM-2) progeny, BioCot-1, BioCot-2 and the control material Coker 310FR. Insect bioassays were conducted using leaf tissue pieces taken from the youngest fully expanded leaf of 120-day-old plants. Four day old insect larvae were used for feeding assays. Larval mortality was recorded daily, from the 2^nd^ to the 6^th^ day after release on the leaf tissues to assess the relative toxicity of the Cry1Ac protein present in different transgenic lines. No mortality was observed in the insect larvae feeding on normal cotton leaf tissues. The consumed leaf area and mortality rate were variable among the tested leaf tissues (Fig 6A and 6B). After one day the mortality rates of larvae feeding on leaf tissues of plants containing events Tg2E-13, TM-2 and their F1 progeny were 16.7%, 6.7% and 36.6%, respectively, whereas no mortality was observed on BioCot-1 or BioCot-2. After two days, 60%, 36.7%, 6.7%, 10% and 100% mortality rates were observed on plants containing Tg2E-13, TM-2, BioCot-1, BioCot-2 and the F1 (Tg2E-13 × TM-2), respectively. A mortality rate of 100% was recorded after three days on leaf tissues of Tg2E-13 and TM-2, whereas BioCot-1 and BioCot-2 caused 100% mortality after four days.

## Discussion

*Bt* strains contain *cry* genes that produce endotoxins specific to some of the major pests of important crop plants [22]. Many *cry* genes have been characterized and tested against major insect pests [51–53]. Expression of a *cry* gene in cotton was first reported in 1987 by Monsanto and the Delta & Pine Land Co. [54]. Subsequently, transgenic cotton containing a *cry1Ac* gene (event Mon 531) was released for commercial use in 1996. However, from the first testing of *Bt* crops in the United States to the present, the development of resistance to Cry toxins in insect pests has remained a major concern [55]. The much subscribed to strategy for delaying resistance development is ‘High dosage/Refuge’ [10, 11, 24]. The success of this strategy depends on using a refuge zone containing non-*Bt* plants susceptible to the pest (s) and *Bt* plants expressing a high concentration of the Cry toxin(s). *Bt* crop should contain no less than 10 times the level of toxin that would kill 99% of the sensitive individuals of an insect pest [10]. Others have suggested even higher dosages of– 25 to 50 times more [24]. A refuge will be most effective if the dose of toxin ingested by insects eating *Bt* plants is high enough to kill all, or nearly all, of the individuals that are heterozygous for resistance [10, 56].

Development of ‘high dosage’ transgenics, however, is not so straightforward. In many instances, high dosage levels lead to abnormal transgenic plants [43, 57]. Additionally, high dosage levels need to be maintained in the plant parts that are preferred pest feeding sites. Finally, if the crop is affected by a pest complex, as cotton is, then multiple toxins or antifeedants will have to be deployed to cover all the pests and delay resistance development.

We have reported here the development of two transgenic events, Tg2E-13 and TM-2, both containing a codon-modified truncated *cry1Ac* gene with the encoded protein targeted to the cytoplasm in event Tg2E-13 and to the plastids in event TM-2. An expression analysis of the Cry1Ac protein in the Tg2E-13 event showed that the Cry1Ac protein levels declined over the growing season, as has been reported for event Mon 531 (in BioCot-1 and BioCot-2) [25–27, 58]. However, event Tg2E-13 is an improvement over the Mon 531 event because the overall expression level is significantly higher (2- to 3-fold) in the leaf tissues, bracts and flower buds. The expression level in the roots is lower, but a high expression level in the roots is not required because lepidopteran pests of cotton do not feed on the underground plant parts.

The spatial and temporal expression of the Cry1Ac protein in event TM-2 was quite different, except that the highest expression level was observed in the leaf tissues of 60-day-old plants, and it declined in the leaf tissues as the plant aged. However, the expression level was 3- to 4-fold higher in the leaf tissues of event TM-2 taken at different times after germination, 3-fold higher in the bract tissues, but lower in the flower buds when compared with similar tissues in the BioCot lines. No protein expression was observed in the root tissues of event TM-2. The quantification of mRNA encoded by the *cry1Ac* gene in the root tissues showed high mRNA levels. This mRNA is translated into a protein that degrades very rapidly, most probably due to its inability to enter leucoplasts with the transit peptide of an *rbcS1b* gene that encodes a protein that is only found in mature chloroplasts.

Import across the plastid envelope membranes is mediated by the Tic and Toc (translocon at the outer/inner envelope membrane of chloroplast) mechanism [59]. A number of Tic and Toc components have been identified. For example, the Toc 159 protein is a major receptor for photosynthetic proteins that are translocated to chloroplast, while Toc 132 is important in the import of non-photosynthetic proteins into plastids. Plastid selectivity in allowing a protein to enter may be determined by the transit peptide of the precursor because swapping transit peptides can result in an interchanged plastid preference [60, 61]. Most of these experiments have been performed by studying the uptake of labeled proteins into isolated plastids at different stages of differentiation. Our results with the event TM-2 clearly have shown that the *cry1Ac* gene is transcribed in the roots but that no protein gets transported to leucoplasts present in the roots. The protein, in the absence of transport to the organelles gets, degraded and therefore, could not be captured in ELISA assays. The selection of the transit peptide is, therefore, significant in expressing transgenes in green or non-green tissues, or both.

Extensive studies have been carried out on field-evolved resistance in insect pests of *Bt* crops [12, 14, 28, 29, 62, 63]. Resistance has been categorized into three classes, incipient resistance (< 1% resistant individuals), early warning of resistance (1–6% resistant individuals), and practical resistance (> 50% resistant individuals and reduced efficacy). Of the two lepidopteran species that attack cotton in the United States, *Heliothis virescens* has remained susceptible but *Heliothis zea* has shown the emergence of resistance. It was clear from the beginning that the toxin dosage in Mon 531 event was high dosage for *H*. *virescens* but just adequate for *H*. *zea*. In India, *H*. *armigera* is still managed by the Mon 531 event and by stacking it with the Mon 15985 event, but *P*. *gossypiella* has developed resistance in some parts of the country [28]. There are also early signs of *P*. *gossypiella* showing resistance to *Bt* cotton in China [29]. Part of the problem could be the lack of a refuge in the fields of smallhold farmers. The problem, in all probability, will increase as Cry toxin levels are low in the floral tissues and developing bolls of transgenic plants currently in the field.

*H*. *armigera* is naturally more resistant to Cry1Ac toxin than *H*. *virescens* [12]. However, Cry1Ac toxin is the most potent toxin for *H*. *armigera*, a major polyphagous pest that affects many crops in India and elsewhere [64, 65]. Therefore, all of the precautions can be taken for prolonging the life of this Cry1Ac protein for the management of *H*. *armigera* and other lepidopteran pests need to be put in place. For the delivery of a high dosage, the two transgenic events described here have higher toxin levels than the current transgenic events in the field. The two events described here can be used to increase the toxin dosage either in the existing transgenics, using the two events singly, or stacking the two in new varieties and hybrids. Because combining the two events, one in which Cry1Ac is in the cytoplasm (event Tg2E-13) and the other in which the protein accumulates in the plastids (event TM-2), delivers a high dosage without effecting plant growth, this approach can be used for other Cry toxins as part of the strategy of developing ‘high dosage’ crops. The two events and their hybrids, in which the two transgene loci are in the homozygous condition, will now be field tested for efficacy against *H*. *armigera*, *P*. *gossypiella and Earias vitella*. Further improvements could take place if transgenes having different action mechanism are stacked along with the Cry toxin-encoding genes [66, 67].

## Supporting Information

S1 Appendix A comparison of the nucleotide sequence of codon-modified *cry1Ac* gene used in this study, codon-modified *cry1Ac*- like gene present in the event Mon531 and the WT *cry1Ac* gene. (DOCX)

S2 Appendix A comparison of amino acid sequence of the Cry1Ac protein contained in the events Tg2E-13 and TM-2 developed in this study, Cry1Ac-like protein in the event Mon 531 and the protein encoded by *WT* gene sequence. (DOCX)

S3 Appendix Primers used for different analyses in the study. (DOCX)

S4 Appendix Southern hybridization analysis of transgenic plants developed with Construct I with a detailed analysis of the event Tg2E-13. (DOCX)

S5 Appendix Southern hybridization analysis of transgenic plants developed with Construct II with a detailed analysis of the event TM-2. (DOCX)

S6 Appendix PCR based differentiation of *cry1Ac* cassettes present in Construct I and Construct II. (DOCX)

S7 Appendix *P* values obtained for different transgenic events at different time points after carrying out one-way ANOVA and Tukey`s HSD analysis. (DOCX)
