# Supplementary material for: High Expression of Cry1Ac Protein in Cotton (Gossypium hirsutum) by Combining Independent Transgenic Events that Target the Protein to Cytoplasm and Plastids
Source: PLoS One. 2016 Jul 8;11(7):e0158603. doi: 10.1371/journal.pone.0158603 (PMC4938423; doi:10.1371/journal.pone.0158603)
Supplement: S1 Appendix — (DOCX) [file pone.0158603.s001.docx]

**S1 Appendix.** Nuclotide sequence comparison by Clustal W (DNASTAR) of the modified *cry1Ac* gene used to develop events Tg2E-13 and TM-2 in this study (denoted as cry1Ac), *cry1Ac*-like gene of event Mon531 present in BioCot-1 and BioCot-2 (partial sequence, US patent No. 7368241) denoted as Mon531 and WT *cry1Ac* gene as described by Adang et al 1987(Accession No. M11068) denoted as Adang.
