# Supplementary material for: High Expression of Cry1Ac Protein in Cotton (Gossypium hirsutum) by Combining Independent Transgenic Events that Target the Protein to Cytoplasm and Plastids
Source: PLoS One. 2016 Jul 8;11(7):e0158603. doi: 10.1371/journal.pone.0158603 (PMC4938423; doi:10.1371/journal.pone.0158603)
Supplement: S2 Appendix — (DOCX) [file pone.0158603.s002.docx]

**S2 Appendix.** Amino acid sequence comparison by Clustal W (DNASTAR) of the Cry1Ac protein encoded in the events Tg2E-13 and TM-2 developed in this study – denoted as cry1Ac; Cry1Ac-like protein encoded in the event Mon531 (present in BioCot-1 and BioCot-2), denoted as Mon531 and amino acid sequence of Cry1Ac described by Adang et al 1987 - denoted as Adang.
