# Supplementary material for: High Expression of Cry1Ac Protein in Cotton (Gossypium hirsutum) by Combining Independent Transgenic Events that Target the Protein to Cytoplasm and Plastids
Source: PLoS One. 2016 Jul 8;11(7):e0158603. doi: 10.1371/journal.pone.0158603 (PMC4938423; doi:10.1371/journal.pone.0158603)
Supplement: S3 Appendix — (DOCX) [file pone.0158603.s003.docx]

| S. No. | Primer name | Description | Primer sequence |
| --- | --- | --- | --- |
| **Primers used for ‘genome walking’ reactions** | | | |
| 1 | Genome walking outer primer | Adaptor specific outer primer | 5’- GAG CGA AGA AGG TAA TCT ACT TCC TTA-3’ |
| 2 | Genome walking inner primer | Adaptor specific inner primer | 5’- AAG GTA ATC TAC TTC CTT AGC CAA CTG-3’ |
| 3 | cry1Ac_F1 | Walk Primary PCR *cry1Ac* | 5’-AAT GCA TCC CAT TGA GAT GGA CGA AA -3’ |
| 4 | cry1Ac_F2 | Walk Secondary PCR *cry1Ac* | 5’-TGT GTC AAG GAC AAG GAG ATG TCC AT-3’ |
| 5 | nptII_R1 | Walk Primary PCR *nptII* | 5’-CAC ATT ATA CGA GCC GGA AGC ATA AAG T -3’ |
| 6 | nptII_R2 | Walk Secondary PCR *nptII* | 5’-AAA CCT GTC GTG CCA GCT GCA TTA TT-3’ |
| **Primers used for event Tg2E-13 and TM-2 specific amplification** | | | |
| 7 | TG_rev1 | three gene cassette specific primer | 5’-TAATGCGTATGACGAACGCAGTGA -3’ |
| 8 | TM_rev1 | two gene cassette specific primer | 5’-CTTCAAAGCAAGTGGATTGATGTGATATCT-3’ |
| 9 | cry1Ac_F3 | *cry1Ac* specific primer | 5’-AAT GCA TCC CAT TGA GAT GGA CGA AA -3’ |
| **Primers used for ‘real time’ PCR analysis** | | | |
| 11 | cry1Ac_real_F | Real time Forward Primer | 5’-CGC GAG GAA ATG CGT ATT CAA T -3’ |
| 12 | cry1Ac_real_R | Real time Reverse Primer | 5’-ACA ATG GGA TAG CTG TGG TCA AG-3’ |
| 13 | cry1Ac probe | Probe for *cry1Ac* real time | 5’-TCA ACG ACA TGA ACA GCG -3’ (6-FAM MGBNFQ) |
| 14 | 18S real time assay mix (ABI- assay no- Hs99999901_s1) | | |

**S3 Appendix.** Primers used for different analyses in this study.
