# Supplementary material for: High Expression of Cry1Ac Protein in Cotton (Gossypium hirsutum) by Combining Independent Transgenic Events that Target the Protein to Cytoplasm and Plastids
Source: PLoS One. 2016 Jul 8;11(7):e0158603. doi: 10.1371/journal.pone.0158603 (PMC4938423; doi:10.1371/journal.pone.0158603)
Supplement: S4 Appendix — (DOCX) [file pone.0158603.s004.docx]

**S4 Appendix.** Southern hybridization analysis of transgenic plants developed with Construct I with a detailed analysis of the event Tg2E-13.

To determine the copy number integrated in the genome of transgenic plants developed with Construct I, genomic DNA from 25 lines was digested with *Eco*RI and blots were sequentially hybridized to probes encompassing the *cry1Ac* (MB probe), *nptII* (LB probe) and *cry1C* (RB probe) genes as shown in Fig A. Hybridization signals with *nptII* and *cry1C* represent the junction fragments towards the LB and the RB of the T-DNA construct, respectively. Hybridization with *cry1Ac* gene was expected to show a fragment at ~2.2 kb in all the lines. A large number of lines were observed to have multiple copy integrations when probed with the *cry1C* probe. Multiple copy insertions were also observed in hybridizations with the *nptII* probe. Hybridization with the *cry1Ac* probe gave the expected fragment size of ~2.2 kb in most of the lines (20 out of 25), no hybridization signal was observed in 4 of the lines. Some of the lines also contained bands of larger sizes which could have resulted from rearrangement of T-DNA during its integration in the plant genome.

Two lines - Tg2E-13 and Tg2H-8 showed a single band hybridization signal with the *nptII* gene. As Tg2H-8 line had very low expression of the Cry1Ac protein, it was not analyzed further. A detailed Southern analysis was carried out for the event Tg2E-13. The line showed no hybridization signal with the *cry1C* probe. This indicated that the *Eco*RI site located in the FMVde promoter had been deleted and the fragment observed is based on the second *Eco*RI site being located - in the genomic DNA. To confirm deletion of the *cry1C* expression cassette from the event Tg2E-13, genomic DNA isolated from the T1 progeny of Tg2E-13 was digested with three different restriction enzymes *Eco*RI, *Nco*I and *Hind*III and DNA blots were sequentially hybridized to probes specific to *cry1Ac* (MB probe), *nptII* (LB probe) and *cry1C* (RB probe) (Fig A). Hybridization with *cry1Ac* probe gave the expected fall out of sizes 2.2 kb, 3.3 kb and 2.7 kb when genomic DNA was digested with enzyme *Eco*RI, *Nco*I and *Hin*dIII, respectively. Hybridization with *nptII* gene fragment, expected to give a fall out of ~1.1 kb on *Nco*I digestion gave the expected size hybridization. Fragments corresponding to the left border junction with *Eco*RI and *Hin*dIII digestion, as observed in earlier Southern hybridizations were also confirmed. Hybridization with *cry1C* probe showed no signal (Fig B). Therefore, the event Tg2E-13 did not carry any sequence of the *cry1C* gene. Southern hybridization clearly showed that event Tg2E-13 contained a single copy of the *cry1Ac* gene driven by a truncated FMV promoter and the marker gene.

**Fig** **S4 (A**) Diagrammatic representation of construct Pnos-*nptII*-ocspA::FMVde-Ωleader-*cry1Ac*-35SpA::35Sde-Ωleader-*cry1C*-35SpA used to develop the event Tg2E-13 showing the placement of the probes used for Southern hybridization; **(B)** Genomic DNA of the progeny of event Tg2E-13 was digested with *Eco*RI, *Nco*I or *Hin*dIII and hybridized to LB, MB and RB probes. Lane 1 is λ-DNA digested with *Hin*dIII used as a DNA size marker. Lane 2 has DNA from untransformed Coker 310 FR.
