# Supplementary material for: High Expression of Cry1Ac Protein in Cotton (Gossypium hirsutum) by Combining Independent Transgenic Events that Target the Protein to Cytoplasm and Plastids
Source: PLoS One. 2016 Jul 8;11(7):e0158603. doi: 10.1371/journal.pone.0158603 (PMC4938423; doi:10.1371/journal.pone.0158603)
Supplement: S5 Appendix — (DOCX) [file pone.0158603.s005.docx]

**S5 Appendix.** Southern hybridization analysis of transgenic plants developed with Construct II with a detailed analysis of the event TM-2.

To determine the copy number integrated in the genome of transgenic events developed with Construct II, genomic DNA from 22 T0 putative transgenics with normal phenotype were digested with *Hind*III for LB integration analysis using *nptII* sequences as a probe for Southern hybridization. For LB analysis, Southern hybridization was carried out with a ~1.1 kb long *Nco*I fragment encompassing the *nptII* expression cassette was used as the probe (Fig A). This probe should hybridize to *nptII* gene cassette along with some genomic DNA towards the LB of the T-DNA. The blot was again probed with a ~ 1.8 kb *Nco*I-*Bam*HI *cry1Ac* gene fragment to analyze the junction fragment towards the RB. Most of lines showed multiple copies of T-DNA on both the borders. While all the transgenic lines showed hybridization signal with the *nptII* gene specific probe, three lines out of twenty two lines did not show any band following hybridization with the *cry1Ac* gene. Only one line (TI-1) was observed to contain a single copy integration of the transgene at both the borders. One of the lines - TM-2 was found to show a single copy insertion (a single band) at the RB using the *cry1Ac* specific probe and two copy insertions at the LB using the *nptII* specific probe. Four lines RA-2, RD-8, VB-4, TD-2 and TK-9 were found to be two copy insertions at both the borders. Two lines (VB-8 and TK-5) showed a single copy insertion with the LB (*nptII*) probe and multiple copies with the RB (*cry1Ac*) probe. Event TM-2 was selected for further characterization based on the above analysis.

Integration pattern of the T-DNA was studied in the progeny of the event TM-2. Genomic DNA isolated from the progeny plants of event TM-2 were digested with three different restriction enzymes *Ssp*I, *Hind*III and *EcoR*V. DNA blots were sequentially hybridized to probes either encompassing the *cry1Ac* gene (RB probe), the *nptII* gene (LB probe) or 35S promoter sequence (RB probe) as shown in Fig A. All the progeny plants tested showed similar hybridization pattern (Fig B). When the *Ssp*I digested DNA was probed with the *cry1Ac* gene sequence - one band was observed, while two bands were observed when the *Hind*III digested DNA blots were probed with the *nptII* gene specific probe. Hybridization pattern indicated that two *nptII* sequences were present at the flanks and the *cry1Ac* gene sequences in all probability were present in the middle. To confirm this, *EcoR*V digested genomic DNA was probed either with the *cry1Ac* gene probe or with the *nptII* gene probe. *EcoR*V separates the integrated gene cassettes from each other by separating 35S promoter sequence from rest of the cassette. Again, two bands of the same sizes were observed when *EcoR*V digested genomic DNA was probed with either *cry1Ac* or *npt*II gene sequences whereas only one band of ~5kb was observed when the same blots were re-probed with the CaMV35S promoter probe. After analysis of the band sizes, it was concluded that two T-DNA expression cassettes have integrated at a single locus in an inverted fashion.

**Fig S5. (A)** Diagrammatic representation of construct Pnos-*nptII*-ocspA::35Sde-synJUTR-TP-*cry1Ac*-35SpA used to develop the event TM-2 showing the location of the probes used for Southern analysis; **(B)** Genomic DNA of progeny of the event TM-2 was digested with three different restriction enzymes *Ssp*I, *Hin*dIII and *EcoR*V. *Ssp*I and *Hin*dIII digested DNA was used for hybridization to the RB (*cry1Ac*) and LB (*nptII*) probes, whereas *EcoR*V digested DNA was used for hybridization to 35S, *nptII* and *cry1Ac* gene probes. Lane 1 is λ-DNA digested with *Hin*dIII used as a DNA size marker. Lane 2 has DNA from untransformed cotton Coker310 FR.
