# Supplementary material for: High Expression of Cry1Ac Protein in Cotton (Gossypium hirsutum) by Combining Independent Transgenic Events that Target the Protein to Cytoplasm and Plastids
Source: PLoS One. 2016 Jul 8;11(7):e0158603. doi: 10.1371/journal.pone.0158603 (PMC4938423; doi:10.1371/journal.pone.0158603)
Supplement: S6 Appendix — (DOCX) [file pone.0158603.s006.docx]

**S6 Appendix.** PCR based differentiation of *cry1Ac* cassettes present in Construct I and Construct II.

PCR forward primers were designed from the *cry1Ac* gene (cry1Ac F1, S3 Appendix). Reverse primers was designed from the FMV promoter sequence to make the amplification specific to Construct I (TG_rev1, S3 Appendix). For Construct II, reverse primer was designed from the *rbcS1b* transit peptide region (TM_rev1, S3 Appendix). Location of the primers for the specific amplification of the two Constructs have been shown in Fig A and B. Both the sets of primers were used to amplify the target region in the parents — Tg2E-13 and TM-2 so as to check their specificity and were found to be highly specific and therefore could be used for identifying plants that contained both the Constructs.

Amplification specific to Construct II (event TM-2) has been represented in Fig C whereas event Tg2-13 specific amplification pattern has been shown in Fig D. For the development of F1 plants, a homozygous line containing the event Tg2E-13 was crossed with TM-2 event in which the *cry1Ac* cassette was present in a hemizygous condition. As a consequence, all the transgenics contained Tg2E-13 (Construct I) specific band while Construct II specific band was present only in ~50% of the F1 plants that were tested for the presence of both the gene cassettes (Fig C and D). Only those F1 plants were analyzed for expression levels of the Cry1Ac protein that contained both the gene cassettes.

**Fig S6. Construct I and II specific amplifications in the parents and F1 progenies of a cross between transgenic events Tg2E-13 (Construct I) and TM-2 (Construct II). Fig A and B show location of the primers. Fig C and D show the amplification patterns**. Fig A shows location of the primers for Construct II specific amplification. Fig B shows location of the primers for Construct I. Fig C shows that F1 progeny plants of Tg2E-13 and TM-2 are segregating for Construct II. Fig D shows that all the progeny plants contain Construct I.
