# Supplementary material for: High Expression of Cry1Ac Protein in Cotton (Gossypium hirsutum) by Combining Independent Transgenic Events that Target the Protein to Cytoplasm and Plastids
Source: PLoS One. 2016 Jul 8;11(7):e0158603. doi: 10.1371/journal.pone.0158603 (PMC4938423; doi:10.1371/journal.pone.0158603)
Supplement: S7 Appendix — (DOCX) [file pone.0158603.s007.docx]

**S7 Appendix.** *P* values obtained for different transgenic events at different time points after carrying out one-way ANOVA and further analyzing data using Tukey`s HSD analysis. *P* values <0.05 were considered as significantly different.

| Year | Age of plants | Name of events | P | Year | Age of plants | Name of events | p |
| --- | --- | --- | --- | --- | --- | --- | --- |
| 2014 | 20 d (leaf tissues) | Tg2E-13 Versus BioCot-2 | 0.7103691 | 2015 | 30 d (leaf tissues) | Tg2E-13 Versus BioCot-2 | 0.5298960 |
|  |  |  |  |  |  | TM-2 versus BioCot-2 | 0.0416787 |
|  |  | TM-2 versus BioCot-2 | 0.0000168 |  |  | TM-2 versus Tg2E-13 | 0.5699177 |
|  |  |  |  |  |  | Tg2E-13 versus F1 | 0.0001776 |
|  |  | TM-2 versus Tg2E-13 | 0.0001175 |  |  | TM-2 versus F1 | 0.0052634 |
|  |  |  |  |  |  | F1 versus BioCot-2 | 0.0000062 |
|  | 60 d (leaf tissues) | Tg2E-13 Versus BioCot-2 | 0.0002927 |  | 60 d (leaf tissues) | Tg2E-13 Versus BioCot-2 | 0.0052718 |
|  |  | TM-2 versus BioCot-2 | 0.0000081 |  |  | TM-2 versus BioCot-2 | 0.0006009 |
|  |  |  |  |  |  | TM-2 versus Tg2E-13 | 0.8688246 |
|  |  | TM-2 versus Tg2E-13 | 0.2386442 |  |  | Tg2E-13 versus F1 | 0.0662076 |
|  |  |  |  |  |  | TM-2 versus F1 | 0.3533270 |
|  |  |  |  |  |  | F1 versus BioCot-2 | 0.0000009 |
|  | 90 d (leaf tissues) | Tg2E-13 Versus BioCot-2 | 0.0000996 |  | 100 d (leaf tissues) | Tg2E-13 Versus BioCot-2 | 0.0122641 |
|  |  |  |  |  |  | TM-2 versus BioCot-2 | 0.0962580 |
|  |  | TM-2 versus BioCot-2 | 0.0152798 |  |  | TM-2 versus Tg2E-13 | 0.8573378 |
|  |  |  |  |  |  | Tg2E-13 versus F1 | 0.0013919 |
|  |  | TM-2 versus Tg2E-13 | 0.0919103 |  |  | TM-2 versus F1 | 0.0001519 |
|  |  |  |  |  |  | F1 versus BioCot-2 | 0.0000032 |
|  | 120 d (leaf tissues) | Tg2E-13 Versus BioCot-2 | 0.0059903 |  | 120 d (leaf tissues) | Tg2E-13 Versus BioCot-2 | 0.0006313 |
|  |  |  |  |  |  | TM-2 versus BioCot-2 | 0.7273015 |
|  |  | TM-2 versus BioCot-2 | 0.1794402 |  |  | TM-2 versus Tg2E-13 | 0.0103632 |
|  |  |  |  |  |  | Tg2E-13 versus F1 | 0.2555899 |
|  |  | TM-2 versus Tg2E-13 | 0.3217205 |  |  | TM-2 versus F1 | 0.0000925 |
|  |  |  |  |  |  | F1 versus BioCot-2 | 0.0000067 |
|  | Flower bud | Tg2E-13 Versus BioCot-2 | 0.0000520 |  | Flower bud | Tg2E-13 Versus BioCot-2 | 0.0006709 |
|  |  |  |  |  |  | TM-2 versus BioCot-2 | 0.0000003 |
|  |  | TM-2 versus BioCot-2 | 0.0001096 |  |  | TM-2 versus Tg2E-13 | 0.0000000 |
|  |  |  |  |  |  | Tg2E-13 versus F1 | 0.9511016 |
|  |  | TM-2 versus Tg2E-13 | 0.0000000 |  |  | TM-2 versus F1 | 0.0000000 |
|  |  |  |  |  |  | F1 versus BioCot-2 | 0.0033600 |
|  | Bracts | Tg2E-13 Versus BioCot-2 | 0.0000096 |  | Bracts | Tg2E-13 Versus BioCot-2 | 0.0000021 |
|  |  |  |  |  |  | TM-2 versus BioCot-2 | 0.0000022 |
|  |  | TM-2 versus BioCot-2 | 0.0000001 |  |  | TM-2 versus Tg2E-13 | 0.9999999 |
|  |  |  |  |  |  | Tg2E-13 versus F1 | 0.0021265 |
|  |  | TM-2 versus Tg2E-13 | 0.0371200 |  |  | TM-2 versus F1 | 0.0020260 |
|  |  |  |  |  |  | F1 versus BioCot-2 | 0.0000000 |
|  | Root | Tg2E-13 Versus BioCot-2 | 0.0001702 |  |  |  |  |
|  |  | TM-2 versus BioCot-2 | 0.0004906 |  |  |  |  |
|  |  | TM-2 versus Tg2E-13 | 0.0000000 |  |  |  |  |
|  | Cotyledon | Tg2E-13 Versus BioCot-2 | 0.0001702 |  |  |  |  |
|  |  | TM-2 versus BioCot-2 | 0.0004906 |  |  |  |  |
|  |  | TM-2 versus Tg2E-13 | 0.0000000 |  |  |  |  |
